# Prevalence and Predictors of Gastrointestinal Dysmotility in Patients with Hypermobile Ehlers-Danlos Syndrome: A Tertiary Care Center Experience

**DOI:** 10.7759/cureus.7881

**Published:** 2020-04-29

**Authors:** Mohammad Alomari, Asif Hitawala, Pravallika Chadalavada, Fahrettin Covut, Laith Al Momani, Shrouq Khazaaleh, Falgun Gosai, Suleiman Al Ashi, Ashraf Abushahin, Alison Schneider

**Affiliations:** 1 Internal Medicine, Cleveland Clinic Foundation, Cleveland, USA; 2 Internal Medicine, Cleveland Clinic - Fairview Hospital, Cleveland, USA; 3 Oncology, Cleveland Clinic - Fairview Hospital, Cleveland, USA; 4 Internal Medicine, East Tennessee State University, Johnson City, USA; 5 Internal Medicine, New York Medical College, New York, USA; 6 Gastroenterology, Cleveland Clinic Florida, Weston, USA

**Keywords:** ehlers-danlos syndrome, dysmotility, fibromyalgia, irritable bowel syndrome, postural orthostatic tachycardia syndrome

## Abstract

Introduction

Ehlers-Danlos syndrome (EDS), specifically the hypermobility type (hEDS), is associated with a variety of gastrointestinal (GI) conditions. This study aims to evaluate the prevalence of and factors associated with gut dysmotility in patients with hEDS.

Methods

This is a retrospective study of hEDS patients conducted at the Cleveland Clinic's Center for Personalized Genetic Healthcare between January 2007 and December 2017. Demographics, GI motility testing, endoscopic, and imaging data were extracted from the patients’ charts.

Results

A total of 218 patients with hEDS were identified. Among them, 136 (62.3%) patients had at least one GI symptom at the time of EDS diagnosis. Motility testing was performed and reported in 42 (19.2%) patients. Out of them, five (11.9%) had esophageal dysmotility, 18 (42.8%) had gastroparesis, five (11.9%) had small bowel/colon altered transit time, and four (9.5%) had global dysmotility. In univariable analysis, patients with postural orthostatic tachycardia syndrome (POTS) [odds ratio (OR): 8.88, 95% CI: 3.69-24.9, p<0.0001], fibromyalgia (OR: 4.43, 95% CI: 2.04-10.1, p=0.0002), history of irritable bowel syndrome (OR: 5.01, 95% CI: 2.31-11.2, p<0.0001), and gastroesophageal reflux disease (OR: 3.33, 95% CI: 1.55-7.44, p=0.002) were more likely to be diagnosed with GI dysmotility. On multivariable analysis, only POTS (OR: 5.74, 95% CI: 2.25-16.7, p=0.0005) was significantly associated with an increased likelihood of GI dysmotility.

Conclusions

This study suggests that GI symptoms are relatively common among patients with hEDS. Of the patients tested for dysmotility, 76.2% were found to have some form of dysmotility. POTS was found to be an independent predictive factor for GI dysmotility.

## Introduction

Ehlers-Danlos syndromes (EDS) is a rare, genetically heterogeneous connective tissue disorder with an overall prevalence of 1/5,000. It has several distinct features, including skin hyperextensibility, joint hypermobility, atrophic scarring, and generalized tissue fragility [1]. The pathophysiology behind most types of EDS involves inherited alterations in genes affecting the synthesis and processing of different forms of collagen. Most of these are associated with defects in fibrillar collagen types I, III, and V. The new international EDS classification published in 2017 defines 13 subtypes of EDS, with hypermobile EDS (hEDS) being the most common subtype [2-4]. The clinical presentation of each EDS subtype is extremely variable, and misdiagnosis leads to a significant burden on management strategies for these patients. Interestingly, a recent survey by the European Organization for Rare Diseases (EURORDIS) has shown that among patients with 16 rare genetic disorders, those with EDS have the longest delay in the diagnosis, often requiring consultation of up to 20 medical specialists [5]. This, consequently, has a considerable impact on the quality of life of these patients [6].

EDS is considered a multisystem disorder, and its effects on connective tissue can involve the entire gastrointestinal (GI) tract in terms of both structure and function. Prior studies have shown that EDS, especially the hEDS subtype, is associated with a variety of GI symptoms and functional GI disorders. Studies have shown an increased prevalence of irritable bowel syndrome (IBS) in these patients when compared to age-matched controls [7]. In addition, the hEDS subtype appears to have more symptoms of dyspepsia (postprandial fullness) and heartburn than other subtypes [8]. Other symptoms reported with higher prevalence include nausea, abdominal pain, diarrhea, chronic constipation, and pelvic floor dyssynergia [9-14].

GI dysmotility has also been reported in patients affected by hEDS [7,11,15]. A few theories that have been put forward as possible mechanisms behind the intestinal dysmotility in EDS and those include autonomic neuropathy, mixed enteric neuro-myopathy, and alterations in the composition of the extracellular matrix in which the other components of the gut wall are embedded [11]. Hence, it can potentially affect any part of the GI tract. Although the association between hEDS and GI symptoms has been described, data on specific GI motility disorders in this subtype is limited. The primary aims of this study are to describe the prevalence and outcomes of GI motility disorders in patients with hEDS as well as to identify the risk factors that may be associated with dysmotility.

## Materials and methods

**Figure 1:**
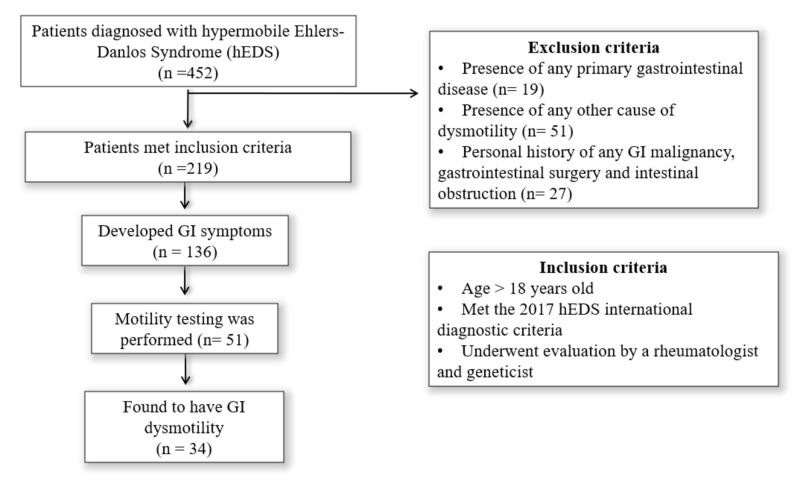
Flow diagram of patients with inclusion and exclusion criteria GI: gastrointestinal

This was a single-center retrospective study. We performed chart review using International Classification of Diseases, Ninth Revision (ICD-9) code 756.83 and International Classification of Diseases, Tenth Revision (ICD-10) code Q79.6 to identify patients diagnosed with hEDS (formerly termed “Ehlers-Danlos Syndrome, hypermobility type”) at the Cleveland Clinic's Center for Personalized Genetic Healthcare between January 2007 and December 2017. Our patients had been originally diagnosed with a combination of Brighton criteria and Villefranche nosology, which created a heterogeneous sample of hypermobility syndrome and hEDS. A thorough review of the electronic medical records was performed for all patients, and the data extracted was used to diagnose hEDS based on the new 2017 international criteria for hEDS [4]. Mostly, the criteria above were assessed for each patient manually (since most of our patients were diagnosed before 2017) in order to identify patients with true hEDS. All the included patients were diagnosed by either a rheumatologist and/or a geneticist. They were then referred to a gastroenterologist when deemed necessary. Notably, most of the included IBS patients were diagnosed based on the ROME III criteria. However, patients with IBS diagnosis after May 2016 were mainly diagnosed using the ROME IV criteria. GI dysmotility was evaluated by one or more of the following methods: esophageal manometry, gastric emptying studies, small/large bowel transit time, GI series, evacuatory studies including anorectal manometry, defecography, and/or wireless capsule motility testing (SmartPill™, Medtronic Inc, Minneapolis, MN). The range of the mean ±SD of the control group was defined as the normal range for GI motility. Patients with a primary GI disease such as inflammatory bowel disease, GI malignancy, a personal history of GI surgery, and patients who did not fulfill the 2017 hEDS diagnosis criteria as well as those diagnosed with any other cause of gut dysmotility like diabetes mellitus were excluded. The study protocol was approved by the institutional review board of the Cleveland Clinic Foundation. The study flow chart with both inclusion and exclusion criteria is illustrated in Figure 1.

Univariable and multivariable logistic regression analyses were performed to identify the predictive factors for gut dysmotility. Predictive factors with a statistical significance on univariable logistic regression analysis (p<0.05) were selected for multivariable analysis. The median follow-up time was calculated using the reverse Kaplan-Meier method [16]. Cumulative incidence was calculated using death as a competing risk. All statistical calculations were made using R statistical software version 3.4.0 (R Foundation for Statistical Computing, Vienna, Austria).

## Results

A total of 452 newly diagnosed hEDS patients were identified, of which 234 patients did not meet the inclusion criteria for reasons noted above. Thus, there were 218 patients included in the final analysis. Patient and disease characteristics are listed in Table 1.

**Table 1:** Patient and disease characteristics *Percentage is calculated based on the total number of patients tested **Percentage is calculated based on the total number of patients with gastrointestinal dysmotility BMI: body mass index; POTS: postural orthostatic tachycardia syndrome; IBS: irritable bowel syndrome

Characteristics	
Age, year, median (range)	32.3 (18.1–69.1)
BMI, kg/m2, median (range)	25.4 (15.0–57.1)
Gender, n (%)	
Male	20 (9.2)
Female	198 (90.8)
Ethnicity, n (%)	
White	195 (89.5)
Black	7 (3.2)
Other	16 (7.3)
Smoking history, n (%)	62 (28.4)
Genetic testing, n (%	63 (28.9)
POTS, n (%)	87 (39.9)
Fibromyalgia, n (%)	78 (35.8)
IBS, n (%)	61 (28.0)
Psychiatric comorbidities, n (%)	
Depression	72 (33.0)
Generalized anxiety disorder	64 (29.4)
Attention deficit disorder	24 (11.0)
Bipolar disorder	13 (6.0)
Any psychiatric comorbidity	105 (48.2)
History of gastroesophageal reflux disease	82 (37.6)
Oropharyngeal dysphagia	3 (1.4)
GI dysmotility, n (%)	
No dysmotility studies performed	176 (80.7)
Dysmotility studies performed	42 (19.2)
No dysmotility*	10 (23.8)
Esophageal dysmotility*	5 (11.9)
Gastroparesis*	18 (42.8)
Small bowel/colon altered transit time*	5 (11.9)
Global dysmotility*	4 (9.5 )
Medication use in patients with GI dysmotility**, n (%)	
Opioid use	12 (37.5)
Prokinetic use	10 (31.2)
Neuroleptic/antipsychotic use	5 (15.6)

The median age at diagnosis was 32.3 years (range: 18.1-69.1) and 199 patients (91%) were female. Postural orthostatic tachycardia syndrome (POTS) was present in 40% of the study population; all those patients were evaluated by a cardiologist and underwent tilt table testing. The median follow-up period from the hEDS diagnosis was 32 months.

Clinical symptoms and diagnosis

One hundred and thirty-six (62.3%) patients had at least one GI symptom at the time of hEDS diagnosis in any clinic. Abdominal pain (49.8%) was the most common symptom, which was followed by nausea (49.5%) and constipation (45.4%) (Table 2).

**Table 2:** Gastrointestinal and non-gastrointestinal manifestations in patients IBS: irritable bowel syndrome

Manifestations	N (%)
Abdominal pain	68 (49.8)
Nausea	67 (49.5)
Constipation	61 (45.4)
Diarrhea	82 (37.6)
Heartburn	78 (35.8)
History of pelvic floor dysfunction	74 (33.9)
IBS	47 (21.6)
IBS – diarrhea subtype	9 (4.1)
IBS – constipation subtype	5 (2.3)
IBS – mixed subtype	6 (2.8)
IBS – unclassified	27 (12.4)
Belching/bloating	59 (27.1)
Vomiting	57 (26.1)
Dysphagia	31 (14.2)
Fecal incontinence	13 (6.0)
History of rectocele	12 (5.5)
Fecal urgency	8 (3.7)
History of rectal prolapse	4 (1.8)

The prevalence of POTS (48% vs. 27%, p=0.002), fibromyalgia (42% vs. 26%, p=0.015), and any psychiatric disease (59% vs. 30%, p<0.0001) were significantly higher among patients with GI manifestations compared to those without any GI symptoms.

Motility testing was performed and reported in 42 (19.2%) patients. Out of them, 10 (23.8%) had no dysmotility, five (11.9%) had esophageal dysmotility, 18 (42.8%) had gastroparesis, five (11.9%) had small bowel/colon altered transit time, and four (9.5%) had global dysmotility. Furthermore, among the 32 patients with dysmotility, 21 patients were diagnosed with GI dysmotility before being diagnosed with hEDS. For them, the median time between diagnosis of GI dysmotility and hEDS was 39 months (range: 0.1-120). The remaining 11 patients were diagnosed with GI dysmotility after the diagnosis of hEDS, with a median time of six months (range: 1-30) (Of note, oropharyngeal dysphagia and pelvic floor dysfunction are reported separately in Tables 1, 2 respectively).

**Figure 2:**
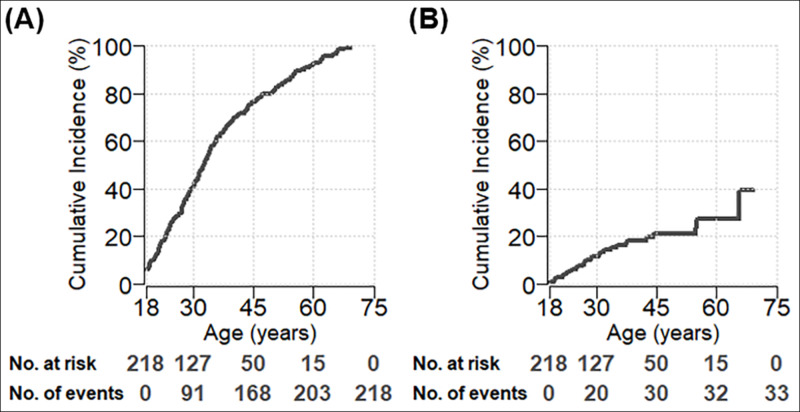
The lifelong cumulative incidence of diagnosis of hypermobile Ehlers-Danlos syndrome and gastrointestinal dysmotility (A): hypermobile Ehlers-Danlos syndrome. (B): gastrointestinal dysmotility

Among the 47 patients who had previously been diagnosed with IBS, eight (17%) were ultimately diagnosed with intestinal dysmotility. Of them, four patients had gastroparesis, two had global dysmotility, one had small bowel/colon altered transit time, and one had esophageal dysmotility. In our cohort, 41.3% of patients were diagnosed with hEDS by the age of 30 (95% CI: 34.7-47.8) and 81.2% by the age of 50 (95% CI: 76.0-86.4) (Figure 2A). The cumulative incidence of GI dysmotility was 11.2% (95% CI: 6.5-15.9) and 21.1% (95% CI: 13.8-28.4) by the ages of 30 and 50 years, respectively (Figure 2B).

One hundred and five patients were diagnosed with at least one psychiatric disease. Of these, 72 had major depressive disorder (MDD), 64 had generalized anxiety disorder (GAD), 24 had attention-deficit hyperactivity disorder (ADHD), 13 had bipolar disorder, two had adjustment disorder, one had conversion disorder, and one had seasonal affective disorder. Furthermore, two patients were diagnosed with anorexia nervosa and had attempted suicide previously.

GI investigations

GI investigations are summarized in Table 3.

**Table 3:** Gastrointestinal investigations *Medications include opiates, prokinetics, and antipsychotics

Investigations	Performed tests, n	Abnormal tests, n	Patients who used medications that can alter gut motility, n*
Videofluoroscopic modified barium swallow	6	3	0
Esophageal manometry	9	5	1
Upper endoscopy	65	36	0
Gastric emptying study	26	18	7
Capsule endoscopy	4	1	1
Colonoscopy	52	31	9
Anorectal manometry/defecography	12	7	4
Smart Pill^TM^	5	3	1
Hydrogen breath test	16	5	4
Upper gastrointestinal series	2	2	1
Antroduodenal manometry	1	0	0

Esophageal Manometry

Esophageal manometry was abnormal in five out of nine patients who were tested. One patient had low resting lower esophageal sphincter (LES) pressure, two patients had hypertensive LES, while the other two patients had ineffective esophageal motility and low amplitude distal esophageal contractions.

Videofluoroscopic Modified Barium Swallow

Videofluoroscopic modified barium swallow was performed in six patients with oropharyngeal dysphagia; only three had evidence of mild to moderate dysphagia.

Upper GI Endoscopy

Sixty-five patients underwent upper GI endoscopy and 36 patients had abnormalities. The most common abnormality was gastritis (18 patients). Ten patients had esophagitis, six had a hiatal hernia, four had duodenal ulcers, three had gastric polyps, three had tortuous esophagus, and two had gastric ulcers.

Gastric Emptying Study

Gastric emptying study was performed in 26 patients and was found abnormal in 18 patients. Six patients had accelerated while 12 had delayed gastric emptying in the four-hour gastric emptying studies. While gastric emptying study was abnormal in 18 patients, a full four-hour test was reported in only eight patients, with a mean gastric retention of 78.5% at hour one, 58.6% at hour two, and 32.6% at hour four.

Colonoscopy

Colonoscopy was abnormal in 31 patients among the 52 who were tested. Fifteen patients had internal hemorrhoids, 12 had polyps, seven had diverticulosis, and one had cecal worms.

Pelvic Floor Studies

Twelve patients underwent anorectal manometry and/or defecography studies; seven had structural and/or functional abnormalities. The most common abnormal findings were paradoxical contraction during defecation in two patients, rectocele in four patients, delayed sensation and urge during volume studies in two patients, and excessive anterior descent with enterocele in one patient.

Wireless Motility Testing

Five patients underwent wireless motility testing (Smart Pill^TM^). One patient had global gut dysmotility and two had isolated delayed colonic transit. The remaining two patients had normal findings.

Other Tests

A total of 16 patients underwent hydrogen breath testing, of which five were interpreted as positive. Upper GI series was performed in two patients and revealed rapid transit time. Antroduodenal manometry was done in one patient and was found to be within normal limits.

Outcomes

In our cohort, 15 (7%) patients were unable to tolerate oral intake and required further intervention. Among 32 patients with confirmed GI dysmotility, 10 (31.2%) required tube feeding, five (15.6%) required total parenteral nutrition (TPN), and 12 (37.5%) required dysmotility-related surgery. Surgeries included laparoscopic jejunostomy for five, bowel resection for three, loop ileostomy for two, and total colectomy for one patient. Additionally, intestinal transplantation was done in one patient due to long-term parenteral nutrition complications. Two out of 12 patients who underwent surgery developed delayed wound healing complicated by incisional hernia.

Additionally, we identified a subset of six patients with severe GI dysmotility; all of them required tube feeding and five of them required additional parenteral nutrition. Among these six patients, four patients underwent dysmotility-related GI surgery, three were diagnosed with small intestinal bacterial overgrowth, four patients had POTS, and one patient had POTS along with mast cell activation syndrome (Table 4).

**Table 4:** Hypermobile Ehlers-Danlos syndrome patients with severe gastrointestinal dysmotility BMI: body mass index; POTS: postural orthostatic tachycardia syndrome; SIBO: small intestinal bacterial overgrowth; PEG: percutaneous endoscopic gastrostomy tube; GI: Gastrointestinal

	Case 1	Case 2	Case 3	Case 4	Case 5	Case 6
Diagnosis	Clinical	Clinical	Clinical	Clinical	Clinical	Clinical
POTS	Present	Present	Present	Present	Present	Absent
SIBO	Absent	Absent	Present	Present	Absent	Present
Age (years)	22	19	26	19	29	24
Sex	Female	Female	Female	Female	Female	Female
BMI (kg/m^2^)	19.67	20.31	24.13	21.55	31.61	22.5
Predominant symptoms	Abdominal pain	Vomiting	Constipation	Vomiting	Bloating	Constipation
Smart Pill™	Small bowel delayed transit	Global dysmotility	Not done	Global dysmotility	Global dysmotility	Not done
GI series	Normal	Normal	Delayed transit	Normal	Normal	Delayed transit
Gastric emptying	Delayed	Delayed	Normal	Delayed	Delayed	Delayed
Mechanical obstruction	No	No	No	No	No	No
Tube feeding	Jejunostomy tube	Corpak	PEG tube	Jejunostomy tube	Jejunostomy tube	PEG tube
Parenteral nutrition	Yes	Yes	No	Yes	Yes	Yes
Surgical intervention	Loop ileostomy	None	Total colectomy	None	Loop ileostomy	Intestinal transplant

On univariable logistic regression analysis, patients with POTS [odds ratio (OR): 8.88, 95% CI: 3.69-24.9, p<0.0001], fibromyalgia (OR: 4.43, 95% CI: 2.04-10.1, p=0.0002), history of IBS (OR: 5.01, 95% CI 2.31-11.2, p < 0.0001), and gastroesophageal reflux disease (OR: 3.33, 95% CI: 1.55-7.44, p=0.002) were more likely to be diagnosed with GI dysmotility. On multivariable logistic regression analysis, only POTS (OR: 5.74, 95% CI: 2.25-16.7, p=0.0005) was significantly associated with an increased likelihood of GI dysmotility (Table 5).

**Table 5:** Univariable and multivariable logistic regression analysis for gastrointestinal dysmotility in patients with hypermobile Ehlers-Danlos syndrome POTS: postural orthostatic tachycardia syndrome; IBS: irritable bowel syndrome; BMI: body mass index; CI: confidence interval

Variables	Univariable analysis		Multivariable analysis	
	Odds ratio (95% CI)	P-value	Odds ratio (95% CI)	P-value
POTS (yes vs. no)	8.88 (3.69–24.9)	<0.0001	5.74 (2.25–16.7)	0.0005
IBS (yes vs. no)	5.01 (2.31–11.2)	<0.0001	2.13 (0.86–5.33)	0.10
Fibromyalgia (yes vs. no)	4.43 (2.04–10.1)	0.0002	2.30 (0.92–5.86)	0.08
Gastroesophageal reflux disease (yes vs. no)	3.33 (1.55–7.44)	0.002	1.68 (0.69–4.13)	0.25
Opioid use (yes vs. no)	2.34 (1.03–5.15)	0.038	2.06 (0.80–5.23)	0.13
BMI (per 1 kg/m^2^ increase)	1.04 (0.99–1.09)	0.14		
Smoking history (yes vs. no)	0.67 (0.25–1.56)	0.38		
Age (per 1-year increase)	0.99 (0.96–1.02)	0.48		

## Discussion

This was a 10-year retrospective study that looked specifically into the prevalence of GI dysmotility in patients with a confirmed diagnosis of hEDS. The major strengths of this study are the large group of patients recruited (n = 218) and the strict inclusion criteria; all patients were evaluated by a rheumatologist and a geneticist and only those who fulfilled the 2017 international hEDS criteria were ultimately included. This study illustrates the high prevalence of GI symptoms and coexisting psychiatric disorders among patients with hEDS, reaching 62.2% and 48.1% respectively. Moreover, POTS was found to be an independent predictive factor for gut dysmotility. Although multiple studies have examined the GI manifestations in hEDS patients, the majority of them neither included GI motility testing nor evaluated the potential need for advanced nutrition such as tube feeding, parenteral nutrition, and/or dysmotility-related GI surgery.

The diagnosis of hEDS is often based entirely on clinical evaluation and family history. The 2017 international EDS classification was used to diagnose hEDS in all of our patients; it takes into account the Beighton score as well as other factors necessary to diagnose hEDS [17-19]. The Beighton score is a useful tool in ascertaining if an individual has generalized joint hypermobility. At the time of diagnosis, the Beighton score for all our patients was ≥5. While in most individuals with hEDS, the gene in which the causative mutation occurred is unknown and unmapped, genetic testing results were available for 63 patients in our study and were negative for the other types of EDS.

This study further supports many findings reported in other studies. The association between hEDS and GI manifestations was first described by Hakim et al. in 2004 [20]. They found that hEDS patients who attended a hypermobility clinic had significantly more GI symptoms compared to age and sex-matched controls (37% vs. 11%). The most common symptoms reported were nausea and abdominal pain. Nevertheless, a causative relationship between abnormalities in connective tissue and GI symptoms had not yet been established at that time. More recently, in a retrospective study of 687 patients done at the Mayo Clinic, Nelson et al. assessed GI motor function in patients with EDS and noted that among those tested, 22.3% had abnormal gastric emptying, 28.3% had abnormal colonic transit, and 60% had rectal evacuation disorder [7]. Most of these patients had hEDS (68.5%) with a Beighton score of ≥4 seen only in 48% of the involved patients. In contrast to the Mayo Clinic study, we used very stringent inclusion criteria to rule out other forms of hypermobility syndromes and confounding GI dysmotility risk factors. Moreover, we examined the procedural management of GI dysmotility to assess disease severity and potential outcomes that were not discussed before.

The most common presenting GI symptoms in our studied patients are abdominal pain (49.8%) followed by nausea (49.5%) and constipation (45.4%). Of all involved patients, 136 (62.3%) patients had at least one GI symptom at the time of diagnosis. This is relatively higher than the observations from the Mayo Clinic study by Nelson et al., which showed 57.5% (271/471) of the patients had at least one GI symptom at the time of diagnosis [7].

There are currently no well-validated national or international management and care guidelines for the management of EDS‐related GI symptoms and disorders. Most of the treatment is geared towards the management of symptoms. Nevertheless, it is crucial to avoid opiates in these patients, and they should be provided with supplemental nutritional support [21,22]. Opiate use of more than three months was documented in 50 patients in the current study, mostly due to chronic abdominal and musculoskeletal pain. This is clinically relevant as opioids can lead to the development of narcotic bowel syndrome, which subsequently increases abdominal pain and constipation. Notably, all patients who underwent motility testing were instructed to stop medications that could affect GI motility (including opiates, prokinetics, neuroleptics/antipsychotics) for 48 hours prior to the study. In order to eliminate the confounding effect of opiates on our results, multivariable analysis adjusting for opiate use was performed. Furthermore, it is important to mention that two of our patients had anorexia nervosa, which can potentially alter gut motility.

In patients with more severe disease, enteral feeding may be necessary to maintain nutritional status and hydration, typically starting with nasogastric or nasojejunal tube trials. Unfortunately, the course is typically progressive, and some patients might require long-term enteral feeding methods. Conversely, parenteral nutrition is usually supportive and occasionally exclusive (TPN). Also, surgery should be performed, if necessary, to provide access for venting/feeding with intestinal transplantation being indicated in selected patients in whom long-term parenteral nutrition cannot be initiated or continued safely [23]. This is reflected in our analysis as six patients with severe GI dysmotility in our study required two or more of the following: tube feeding, parenteral nutrition, and dysmotility-related GI surgery. In our study, patients with severe GI dysmotility requiring TPN or surgery after exhausting available medical options were managed by a multidisciplinary management team involving a rheumatologist, gastroenterologist, nutritionist, autonomic neurologist, and a surgeon with experience in the treatment of GI dysmotility [24].

A systematic review of GI surgeries and related complications in EDS suggests that surgery in patients with EDS is associated with a high risk of complications, which is why preoperative indications should be carefully considered [25]. In our analysis, 20% of the patients who underwent GI surgeries developed postoperative complications, notably incisional hernias and poor wound healing. The inherent dysfunctional collagen synthesis in EDS might affect the structural integrity of supportive structures such as ligaments, blood vessels, and internal organs and can theoretically increase the risk of postoperative complications [26,27].

It was observed in previous studies that patients with hEDS frequently present with persistent GI symptoms and chronic pain and often meet the criteria for fibromyalgia and functional GI disorders such as functional dyspepsia and IBS [28]. Fibromyalgia was seen in 35.8% of hEDS patients in our study, which is a significantly higher prevalence than those observed in Nelson et al. (20.7%) and Fikree et al. (10.5 %) [7,28]. However, the latter used the Wolfe criteria to diagnose fibromyalgia and not the revised version published in 2010, which might have contributed to this discrepancy in results. Furthermore, our study included 105 patients with a history of psychiatric disorder at some point (only five patients were actively on treatment with antipsychotics/neuroleptics at the time of the motility study). Regardless, this should be kept in mind as these medications often interfere with GI motility.

POTS is a disorder of reduced orthostatic tolerance, defined by a heart rate increment of 30 beats/minute or more within 10 minutes of standing or head-up tilt in the absence of orthostatic hypotension [29]. A large cohort study of 163 patients with POTS was evaluated, and 55 (34%) were found to have a normal gastric emptying time, 30 (18%) had a delayed or slowed gastric emptying time, and 78 (48%) were found to have rapid gastric emptying, suggesting that hEDS, autonomic symptoms, and GI symptoms are indeed linked, though the exact mechanism is unknown [30]. This contrasts with our findings as 38% of the patients diagnosed with POTS had delayed gastric emptying while only 14.2% had accelerated gastric emptying. This confirms the complexity and wide heterogeneity of the GI dysautonomia that can manifest in patients with POTS.

This study has some limitations, including the retrospective nature and the lack of a control group for comparison of our observations with age and sex-matched healthy individuals. Also, this was a single-center experience with a significant number of patients who were lost to follow-up during the study duration, and this might have led to underestimating the overall complication rate and the need for advanced nutrition or dysmotility-related GI surgery. Finally, GI motility tests were not systematically performed in our patients and only a minority of them had the test(s) done. Thus, the true prevalence of gut dysmotility could not be established in our cohort. Caution should be exercised while interpreting our findings as we only reported the prevalence of GI dysmotility in the examined cohort. Furthermore, the diagnosis of SIBO using the hydrogen breath test has considerable limitations in the setting of GI dysmotility, which might have misrepresented the true prevalence of SIBO in our cohort. Also, the information on the prevalence of orthopedic issues and other autonomic (e.g., bladder) dysfunction could not be obtained by a mere chart review.

## Conclusions

GI dysmotility among patients with hEDS is not uncommon and possibly underdiagnosed. In our study, POTS was found to be an independent predictive factor for GI dysmotility in patients with hEDS. However, further prospective studies are required to investigate the pathophysiology of these GI manifestations in patients with hEDS in order to improve both the outcomes and the quality of care in this unique and complicated patient population.
